# Nicotinamide Phosphoribosyl Transferase (Nampt) Is Required for *De Novo* Lipogenesis in Tumor Cells

**DOI:** 10.1371/journal.pone.0040195

**Published:** 2012-06-29

**Authors:** Sarah C. Bowlby, Michael J. Thomas, Ralph B. D’Agostino, Steven J. Kridel

**Affiliations:** 1 Department of Cancer Biology, Wake Forest School of Medicine, Winston-Salem, North Carolina, United States of America; 2 Department of Biochemistry, Wake Forest School of Medicine, Winston-Salem, North Carolina, United States of America; 3 Department of Biostatistical Sciences, Wake Forest School of Medicine, Winston-Salem, North Carolina, United States of America; 4 Comprehensive Cancer Center, Wake Forest School of Medicine, Winston-Salem, North Carolina, United States of America; Laurentian University, Canada

## Abstract

Tumor cells have increased metabolic requirements to maintain rapid growth. In particular, a highly lipogenic phenotype is a hallmark of many tumor types, including prostate. Cancer cells also have increased turnover of nicotinamide adenine dinucleotide (NAD^+^), a coenzyme involved in multiple metabolic pathways. However, a specific role for NAD^+^ in tumor cell lipogenesis has yet to be described. Our studies demonstrate a novel role for the NAD^+^-biosynthetic enzyme Nicotinamide phosphoribosyltransferase (Nampt) in maintaining *de novo* lipogenesis in prostate cancer (PCa) cells. Inhibition of Nampt reduces fatty acid and phospholipid synthesis. In particular, short chain saturated fatty acids and the phosphatidylcholine (PC) lipids into which these fatty acids are incorporated were specifically reduced by Nampt inhibition. Nampt blockade resulted in reduced ATP levels and concomitant activation of AMP-activated protein kinase (AMPK) and phosphorylation of acetyl-CoA carboxylase (ACC). In spite of this, pharmacological inhibition of AMPK was not sufficient to fully restore fatty acid synthesis. Rather, Nampt blockade also induced protein hyperacetylation in PC-3, DU145, and LNCaP cells, which correlated with the observed decreases in lipid synthesis. Moreover, the sirtuin inhibitor Sirtinol, and the simultaneous knockdown of SIRT1 and SIRT3, phenocopied the effects of Nampt inhibition on fatty acid synthesis. Altogether, these data reveal a novel role for Nampt in the regulation of *de novo* lipogenesis through the modulation of sirtuin activity in PCa cells.

## Introduction

Nicotinamide adenine dinucleotide (NAD^+^) is central to many cellular processes. Synthesis of NAD^+^ proceeds through multiple pathways. Most tissues synthesize NAD^+^ through the salvage of nicotinamide by Nicotinamide phosphoribosyl transferase (Nampt) [1], [2]. Cancer cells have a high rate of NAD^+^ turnover compared to normal cells [3]. Accordingly, Nampt is essential for the survival of tumor cells. Pharmacological blockade of Nampt reduces viability in multiple types of cancer cells and can inhibit the growth of tumor xenografts *in vivo* [3], [4], [5]. In addition, Nampt overexpression has been observed in several cancers, including prostate, suggesting a key role in tumor biology [6], [7], [8], [9], [10]. Similarly, Nampt expression increases the lifespan of non-transformed mammalian cells [11], [12], [13]. Because of these findings, Nampt is considered a rational target in cancer and several small molecule inhibitors of the enzyme have been developed [3], [14], [15], [16].

It has become apparent that NAD^+^ has multiple roles in cellular metabolism [17]. Several classes of enzymes utilize NAD^+^ as a substrate during their catalytic reactions. Prominent among them are the sirtuin family of protein deacetylates [18]. Sirtuins regulate the acetylation status of proteins, providing a rapid and efficient way for tumor cells to respond to nutrient and energy changes in the environment [19]. Proteomic analyses have identified over 2800 human proteins that can be acetylated [20]. Interestingly, enzymes that participate in metabolic pathways are enriched within the acetylated protein pool [21], [22]. Among them, enzymes in the fatty acid synthesis pathway were specifically identified [22]. Consistent with this, the mammalian acetyl-CoA synthetases (AceCS1, AceCS2) are deacetylated and activated by SIRT1 and SIRT3 [23], [24]. Conversely, SIRT1 and SIRT3 also regulate fatty acid oxidation via multiple acetylation substrates [25], [26], [27]. Further support for the role of sirtuins in fatty acid metabolism is provided by the finding that mice deficient in the *Sirt1* gene also demonstrate increased phosphorylation of AMPK, an enzyme central in the regulation of energy homeostasis and fatty acid metabolism [28]. Collectively, these studies suggest that NAD^+^ metabolism via Nampt and sirtuins may have a role in fatty acid metabolism in cancer cells.

Tumors of multiple origins, including prostate, exhibit a lipogenic phenotype that Is required for proliferation and survival [29]. Increased *de novo* lipogenesis in tumors is achieved through multiple mechanisms, ranging from gene amplification to post-translational modifications [30], [31], [32], [33], [34]. Because of the central role of lipogenesis in tumors, it is important to identify new markers and pathways which facilitate this process to improve our understanding of tumor biology. Because sirtuins regulate fatty acid and lipid metabolism [23], [24], [25], [27], and Nampt is required for sirtuin function [35], we hypothesized that Nampt is also required for fatty acid and lipid metabolism in tumor cells. We demonstrate that inhibition of Nampt and sirtuins reduces fatty acid and lipid synthesis in tumor cells. Although Nampt inhibition is associated with decreased ATP and AMPK activation, in our system the effects on fatty acid synthesis were primarily mediated by SIRT1 and SIRT3. Collectively, these data highlight a novel convergence between NAD^+^ metabolism, sirtuins, and lipogenesis in prostate cancer. As a result, this study provides new insight into metabolic control in tumor cells that could improve targeted therapies for clinical intervention.

## Materials and Methods

### Materials

Antibodies against Acetylated Lysine (AcK), ACC, phospho-ACC (S79), AMPK, phospho-AMPK (T172), SIRT1, SIRT3 and AceCS1 were from Cell Signaling Technologies (Beverly, MA). The antibody against fatty acid synthase (FASN) was from BD Transduction Labs (San Diego, CA, USA). The antibody against β-actin was from Sigma-Aldrich (St. Louis, MO, USA). The antibody against Nampt (PBEF1, visfatin) was from Phoenix Pharmaceuticals (Burlingame, CA, USA). Secondary antibodies for goat-anti-rabbit or goat-anti-mouse were from BioRad (Hercules, CA, USA). The Nampt inhibitor FK866 was provided by the National Institute of Mental Health Chemical Synthesis and Drug Supply Program (Rockville, MD, USA).

### Cell Culture and Drug Treatments

PC-3, DU145, LNCaP, C4-2, Snb-19, and MCF-7 cells were obtained from American Type Culture Collection (ATCC Manassas, VA, USA). 3T3-Src cells were the generous gift of Darren Seals and have been descried previously [36]. Cell culture medium and supplements for tumor cell lines were supplied by Invitrogen (Carlsbad, CA, USA). Normal prostate epithelial cells (PRECs) and media were obtained from Lonza (Switzerland). PCa cell lines were maintained in RPMI 1640 and Snb-19, 3T3-Src, and MCF-7 cells were maintained in high glucose DMEM supplemented with 10% fetal bovine serum (FBS), Penicillin (100 Units/mL), and Streptomycin (100 µg/mL) at 37°C and 5% CO_2_. PRECs were maintained in Clonetics media according to the manufacturer’s instructions. Cells were treated at times and concentrations indicated. FK866 was dissolved in DMSO and vehicle control is 0.1% DMSO for treatments. NAD^+^, nicotinamide mononucleotide (NMN), and nicotinic acid (Na) were obtained from Sigma-Aldrich and dissolved in water. The sirtuin inhibitor Sirtinol and the AMPK inhibitor Compound C were also obtained from Sigma-Aldrich and dissolved in DMSO.

### Western Blotting

Cells were treated as indicated and harvested, washed with cold PBS, and lysed in buffer containing 1% Triton X-100, 20 mM Tris-HCl, pH 8.3 and protease, phosphatase, and deacetylase inhibitors (1 mM sodium orthovanadate and sodium fluoride, 200 nM okadaic acid, 5 µg/mL aprotinin, pepstatin, and leupeptin, 200 µM PMSF, 2 mg/mL Trichostatin A, 100 µM Sirtinol). Protein samples were assayed with BioRad DC reagents, resolved by SDS-PAGE, and transferred to Immobilon-P membrane (polyvinylidene difluoride). Immunoreactive bands were detected by enhanced chemiluminescence (Perkin Elmer Life Sciences, Boston, MA, USA). For mitochondrial fractionation, cells were lysed in hypotonic buffer (20 mM Hepes, 10 mM KCl, 250 mM sucrose, 1.5 mM MgCl_2_ plus protease and phosphatase inhibitors) using a Dounce homogenizer (Pestle B). Lysates were centrifuged at 10,000 g for 20 minutes at 4°C and the pellet was collected and lysed in buffer containing NP-40 alternative (1% w/v), glycerol (10% v/v), 25 mM Hepes (pH 7.9), 100 mM NaCl, and 1 mM EDTA with protease and phosphatase inhibitors. The supernatant was centrifuged 16,000 g for 20 minutes at 4°C and collected as the “cytoplasmic” fraction. The remaining pellet from the cytoplasmic supernatant was resuspended in the NP-40 lysis buffer described above as the “mitochondrial” fraction [37].

### Total NAD, NADP, and ATP Quantification

Total cellular NAD was measured using the EnzyChrom Assay Kit, (Hayward, CA, USA). Cells were seeded in triplicate in 6-well plates (3×10^5^ cells/well). After 48 hours the cells were treated with FK866 for another 48 hours and total NAD was determined according to the manufacturer’s instructions. For total NADP measurements, cells were treated as described above and measurements were performed using the BioVision NADP^+^/NADPH Quantification Kit (Milpitas, California, USA) according to the manufacturer’s instructions. Cellular ATP levels were measured by bioluminescence using *CellTiter-Glo* from Promega (Madison, WI, USA) according to the manufacturer’s instructions. PCa cells were plated at 10^4^ cells/well in Perkin Elmer white polystyrene 96 well culture plates. Luminescence was normalized to DNA content as detected by CyQUANT fluorescent reagent from Invitrogen. Cells for the CyQUANT assay were plated and treated in Costar black-walled 96 well plates (Corning Inc., Corning, NY, USA).

### Cell Death Assays

PCa cells were plated at low density (800 cells per well of 6-well plates) and treated for 24 hours in triplicate, as indicated. Cells were maintained until visible colonies (>40 cells/colony) were formed, then washed with PBS, fixed with methanol, stained with crystal violet, and colonies were quantified by counting as described previously [38]. For Trypan blue exclusion assays, cells were grown in 6-well plates, and treated as indicated. Cells were trypsinized until detached and the reaction was stopped with culture media with serum. All cells were collected and resuspended to equal volume in PBS. Trypan blue reagent was added for 10 minutes before counting. Live and dead cells were counted and quantified as percent of total cells.

### siRNA Knockdown

ON-TARGETplus siRNA oligonucleotide against Nampt (GGUAAGAAGUUU CCUGUUA), SIRT1 (SIRT1.1 GCGAUUGGGUACCGAGAUA, SIRT1.2 GGAUAGGUCCAU AUACUUU) and SIRT3 (SIRT3.1 UUGAGAGAGUGUCGGGCAU, SIRT3.2 GGACCAGACAAAUAGGAUG) and siGENOME Non-Targeting siRNA #2 were designed and synthesized by Dharmacon (Lafayette, CO, USA). The siRNA (100 nM) were transfected into cells with Lipofectamine 2000 according to the manufacturer’s instructions (Invitrogen). After 4 hours the transfection media was replaced with antibiotic-free RPMI plus 10% FBS until cells were harvested for western blot analysis or metabolic labeling.

### Metabolic Labeling

To measure fatty acid synthesis, 10^5^ cells per well were seeded in 24-well plates (n = 4 wells per treatment). Cells were treated with FK866, Compound C, Sirtinol, NAD^+^ metabolites, or combinations thereof for 48 hours. For siRNA knockdown, cells were transfected with siRNA and incubated for 4–5 days. ^14^C-acetate (1 µCi) or ^14^C-choline (1 µCi) was added to each well for 2 hours. Cells were collected, washed, and lysed with hypotonic buffer (20 mM Tris-HCl, 1 mM EDTA, 1 mM DTT, pH 7.5), and lipids were extracted using chloroform/methanol (2∶1) as we have previously described [38]. Newly synthesized lipids were measured by scintillation counting and counts were normalized to total protein.

### Fatty Acid Methyl Ester (FAME) and Phospholipid Analysis

PC-3 cells were seeded at 3×10^5^ cells/well of 6-well plates (n = 3 wells per treatment) and after 48 hours cells were treated with DMSO (0.1%), FK866 (100 nM), or FK866 plus NMN (500 µM) for 48 hours. Cells were trypsinized, collected, and normalized to cell number. Cell pellets were washed with cold 0.9% saline and frozen in liquid nitrogen. Lipids were extracted using the Bligh-Dyer method [39] and the combined fraction was stored at −80°C. For fatty acid analyses, an aliquot was combined with internal standards (pentadecanoic acid (15∶0) and heneicosanoic acid (21∶0)), evaporated under Argon gas, and resuspended in ethanol. KOH (50%) was added to samples and incubated for 1 hour, 60°C. Non-saponifiable lipids were extracted with hexane and discarded. The remaining phase was acidified with 6 M HCl and lipids were recovered with 3 mL of hexane. After evaporating the hexane, methanolic NaOH (0.5 M) was added and samples were incubated for 5 minutes at 100°C. BF_3_ (14%) was added and the solution was heated to 100°C for an additional 5 minutes. After cooling, hexane (2 mL) and saturated aqueous NaCl (4 mL) were added and vortexed. The hexane phase was recovered, evaporated, and the residue was resuspended in 100 µL hexane and samples were transferred to glass autosampler tubes for analysis. The fatty acids were separated and quantified using a DB-Wax column in a TSQ Quantum XLS triple quadrupole mass spectrometer connected to a TRACE Ultra gas chromatograph (GC-MS). Values were represented as mean picomoles of fatty acid per nanomoles of phosphorous plus or minus standard deviation.

For phospholipid analysis, lipids were extracted as described above then resuspended in CHCl_3_:MeOH (1∶1). To normalize samples, total phosphorous levels for each sample were measured from 50 µL aliquots as described by Rouser, *et al.* [40]. Lipids were diluted to 2 nmol/mL lipid phosphorous with CHCl_3_:MeOH including 1% formic acid. Glycerophospholipids were detected using a TSQ Quantum Discovery Max electrospray tandem mass spectrometer equipped with an Agilent 1100 LC. Phosphatidylcholine (PC) molecular species were measured by recording precursors of *m/z* +184.1, phosphatidylethanolamine (PE) by common neutral loss of 141 Da in the positive ion mode, phosphatidylinositol (PI) by measuring by precursors of *m/z* 241 in negative ion mode, and phosphatidylserine (PS) using the common neutral loss of 185 Da as outlined by DeLong, *et al.* [41]. Lipid species were excluded from further consideration if more than 1 of the 3 replicates for any given treatment group was a zero value. Molecular species were quantified as mol% of the class by correcting for carbon isotope and mass-dependent ion throughput. Lipid species are represented according to class and species as the mean picomoles of lipid per nanomoles of phosphorous plus or minus standard deviation. For each species we compared phosphorous levels (pmol/nmol) among groups (DMSO/FK866/FK+NMN) using one-way analysis of variance (ANOVA) models. In order to avoid performing multiple tests with the possibility of finding spurious results, pairwise comparisons were only made between groups if the overall test from the ANOVA was significant. When the overall ANOVA was significant post-hoc pair-wise comparisons were performed between groups using Student’s *t*-tests. Molecular species are defined as XX:Y where XX refers to the sum of the carbons in the constituent fatty acids and Y the total number of double bonds.

### Statistical Analysis

Significance between groups was determined by ANOVA followed by pair-wise Student’s *t*-tests and is defined as p<0.05.

## Results

### Nampt Activity Regulates PCa Cell NAD^+^ Levels and Survival

**Figure 1:**
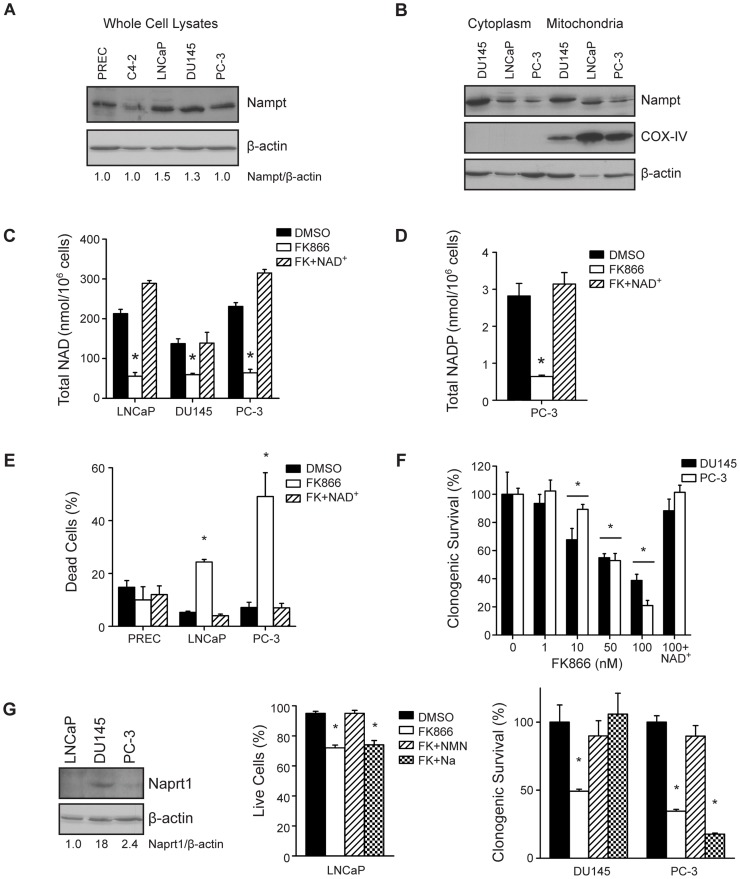
Nampt regulates NAD^+^ levels and survival in PCa cells (A) Nampt protein levels were determined by western blotting in PREC and PCa cells. (B) Nampt localization was also determined in the cytoplasmic and mitochondrial fractions of PCa cells by western blot, with COX-IV as a mitochondrial marker (C) Total NAD levels were measured in PCa cells after 48 hour treatment with vehicle (DMSO 0.1%), or FK866 (100 nM) in the absence or presence of NAD^+^ (100 µM) (*p<0.01). (D) Total NADP levels were measured in PC-3 cells after 48 hour treatment with vehicle (DMSO 0.1%), or FK866 (100 nM) in the absence or presence of NAD^+^ (100 µM) (*p<0.01). (E) The effect of FK866 on survival was assessed by trypan blue exclusion in PREC, LNCaP, and PC-3 cells after 48 hours of treatment. (F) Clonogenic survival of PC-3 and DU145 cells was measured following a dose-response of FK866, or 100 nM FK866 plus NAD^+^ (100 µM) for 24 hours (*p<0.01). (G) Expression of Naprt1 and β-actin were determined by western blot. The ability of NMN and Na to protect cells from FK866 was determined by trypan blue exclusion in LNCaP cells (48 hour treatment) and clonogenic survival in PC-3 and DU145 cells (24 hour treatment).

To investigate the role of Nampt in prostate cancer, protein expression was determined in normal PREC, and tumorigenic C4-2, LNCaP, DU145, and PC-3 cells by western blot. Nampt expression was comparable across all cell lines (Figure 1A). Cell fractionation demonstrated Nampt localization in the cytoplasmic and mitochondrial compartments of prostate tumor cell lines (Figure 1B). Pharmacological blockade of Nampt with the small molecule active site inhibitor FK866 [3] reduced total NAD levels to 26–43% of control levels after 48 hours, and levels were restored by exogenous NAD^+^ (*p<0.01, Figure 1C). Similarly, FK866 reduced total NADP to 23% of control, which was restored by exogenous NAD^+^ (Figure 1D). Although PRECs express Nampt, viability was unaffected by FK866 treatment (Figure 1E). On the other hand, FK866 induced cell death in 25% of LNCaP and in 50% of PC-3 cells (*p<0.01). The addition of exogenous NAD^+^ prevented FK866-induced cell death (Figure 1E). Treatment with FK866 also reduced clonogenic survival of PC-3 and DU145 cells in a dose-dependent fashion, and clonogenic survival was also rescued by exogenous NAD^+^ (*p<0.01, Figure 1F).

In the salvage pathway, Nampt produces nicotinamide mononucleotide (NMN) from nicotinamide. In the *de novo* pathway, Nicotinic acid phosphoribosyltransferase (Naprt1) produces nicotinic acid mononucleotide (NaMN) from nicotinic acid (Na). Western blot analysis demonstrated that Naprt1 was highly expressed in DU145 cells but not PC-3 or LNCaP cells (Figure 1G). Viability was also determined in PCa cells treated with FK866 in the absence or presence of NMN (salvage) or Na (*de novo*). Consistent with the western blot results, in DU145 cells FK866-induced death was rescued by both NMN and Na (*p<0.0001, Figure 1G). On the other hand, survival was only restored by NMN and not Na in LNCaP and PC-3 cells (*p<0.0001). These data indicate that NAD^+^ synthesis is required for the survival of PCa cells and while NAD^+^ salvage through Nampt predominates, the *de novo* pathway is active in at least one cell line.

### Nampt Activity is Required for Fatty Acid Synthesis

**Figure 2:**
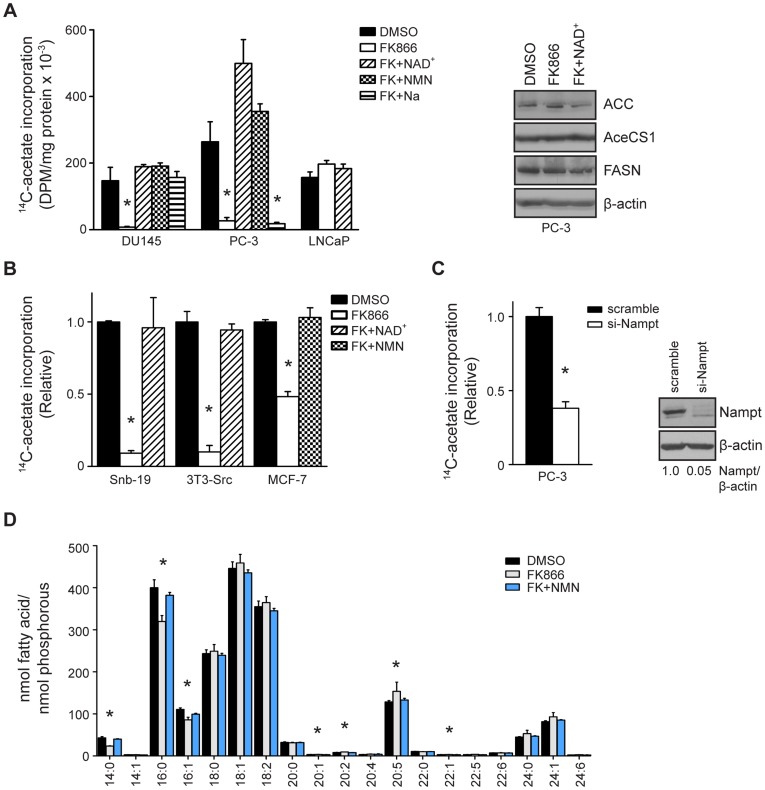
Nampt activity is required for fatty acid synthesis in cancer cells (A) PCa cells were treated with FK866 (100 nM) in the absence or presence of NAD^+^, NMN, or Na for 48 hours and fatty acid synthesis was determined by the incorporation of ^14^C-acetate into the lipid (*p<0.0001). Expression of ACC, AceCS1, FASN, and β-actin was determined by western blot. (B) Fatty acid synthesis was measured in Snb-19 glioblastoma cells, Src-transformed 3T3 fibroblasts, and MCF-7 breast cancer cells as in (A) (*p<0.0001). (C) PC-3 cells were transfected with scrambled or Nampt-targeting siRNA (100 nM) and fatty acid synthesis was assayed 5 days post-transfection (*p<0.0001). (D) PC-3 cells were treated as indicated and the fatty acid profile was determined by GC-MS.

To determine if Nampt is required for the lipogenic phenotype in tumor cells, *de novo* fatty acid synthesis was measured. Treatment of PC-3 and DU145 cells with FK866 reduced fatty acid synthesis, as measured by incorporation of ^14^C-acetate into lipid, by 90% and 95%, respectively (*p<0.0001, Figure 2A). Consistent with the effects on survival, NAD^+^ and NMN restored fatty acid synthesis in both cell lines. Similarly, Na restored fatty acid synthesis in DU145 cells, but not PC-3 cells. Interestingly, treatment with FK866 did not reduce fatty acid synthesis in LNCaP cells, despite the reduction in NAD^+^ levels (Figure 1B). Reduced fatty acid synthesis was not associated with decreased expression of the fatty acid synthesis enzymes ACC, AceCS1, or FASN. Treatment with FK866 also reduced fatty acid synthesis in tumor cells of various origins including Snb-19 glioblastoma cells, Src-transformed 3T3 cells (3T3-Src), and MCF-7 breast cancer cells by 90%, 91%, and 52%, respectively (*p<0.0001, Figure 2B). Co-treatment with NAD^+^ and NMN restored fatty acid synthesis in these cells. Transfection of PC-3 cells with Nampt-specific siRNA also reduced fatty acid synthesis by 62% compared to cells transfected with scrambled siRNA (*p<0.0001, Figure 2C).

Fatty acid methyl ester (FAME) analysis was performed to determine how Nampt inhibition affected the fatty acid profile of PC-3 cells (Figure 2D). Inhibition of Nampt significantly reduced the levels of the *de novo* synthesized 14∶0 and 16∶0 fatty acid species to 57% and 83% of control, respectively, and the 16∶1 fatty acid species to 81% of control (*p<0.05, DMSO versus FK866). Exogenous NMN restored these fatty acids back to control levels. Because *de novo* synthesized fatty acids decreased, the relative levels of the long chain and polyunsaturated fatty acids species subsequently increased. Overall, the data illustrate that blockade of Nampt activity reduces *de novo* fatty acid synthesis.

**Figure 3:**
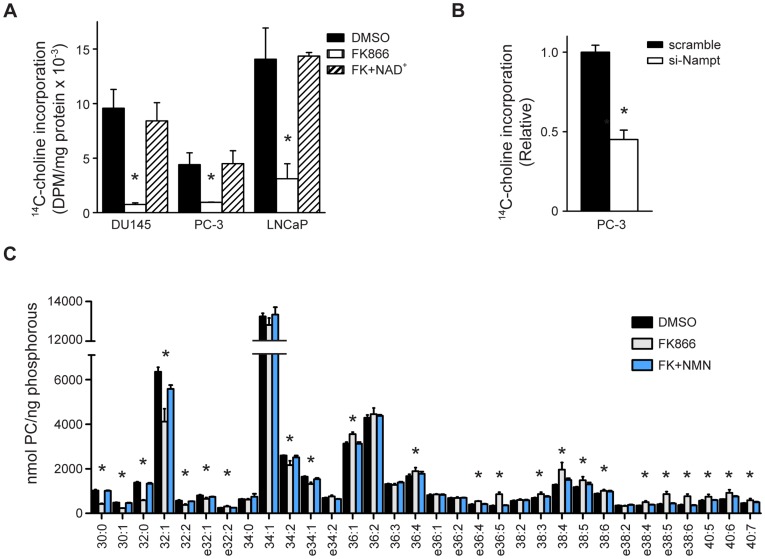
Nampt activity is required for phosphatidylcholine (PC) synthesis in cancer cells (A) PCa cells were treated for 48 hours with vehicle, or FK866 (100 nM) in the absence or presence of NAD^+^ (100 µM) and lipid synthesis was assessed by ^14^C-choline incorporation (*p<0.0001). (B) PC-3 cells were transfected with scrambled or Nampt-targeting siRNA (100 nM) and lipid synthesis was assayed 5 days post-transfection (*p<0.0001). (C) PC-3 cells were treated as indicated and the PC profile was determined by mass spectrometry (*p<0.05).

The effect of Nampt inhibition on lipid metabolism was also assessed by ^14^C-choline incorporation into lipid. Treatment with FK866 reduced incorporation of ^14^C-choline into lipid by 75% in PC-3 cells and 82% in DU145 cells (*p<0.001, Figure 3A). Interestingly, FK866 treatment also reduced incorporation of ^14^C-choline into lipid by 80% in LNCaP cells (*p<0.001). This suggests that fatty acid and lipid metabolism may be differentially regulated by NAD^+^ in LNCaP cells. Therefore, Nampt activity appears to be required for choline incorporation into lipids in PCa cells, independent of fatty acid synthesis. Knockdown of Nampt recapitulated the effects of FK866 on lipogenesis. Transfection of PC-3 cells with Nampt-specific siRNA reduced lipid synthesis by 55% relative to cells transfected with scrambled siRNA (*p<0.0001, Figure 3B).

**Figure 4:**
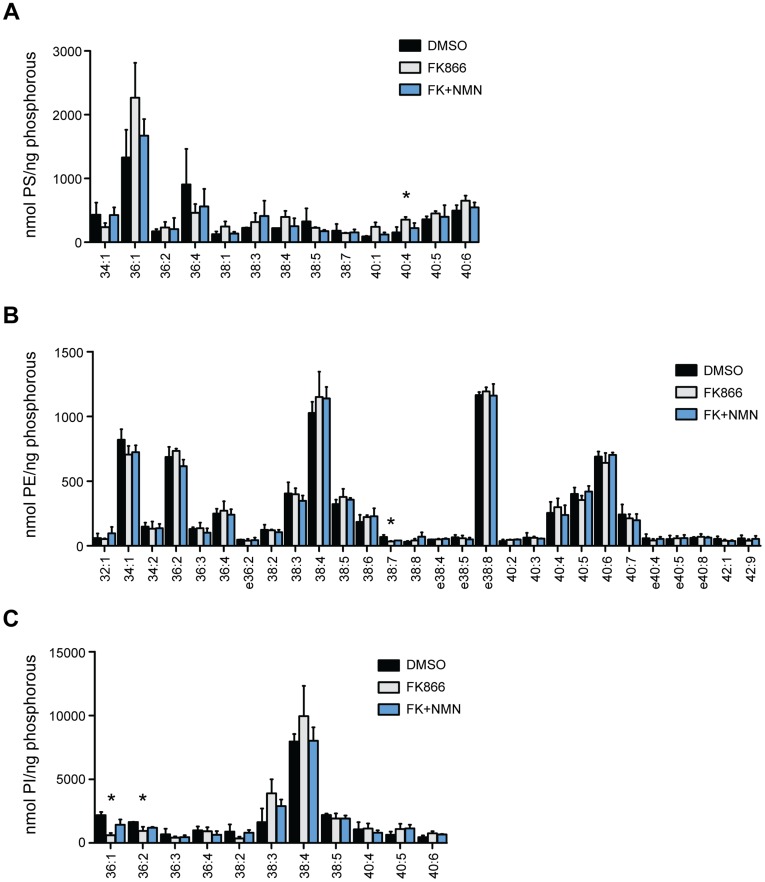
Nampt inhibition does not affect the phosphatidylserine (PS), phosphatidylethanolamine (PE), or phosphatidylinositol (PI) lipid classes. (A–C) PC-3 cells were treated with vehicle, FK866 (100 nM) in the absence or presence of NMN (500 µM) for 48 hours and the PS, PE, and PI profiles were determined by mass spectrometry (*p<0.05).

The cellular phospholipid profile was determined by mass spectrometry in PC-3 cells following Nampt inhibition. Interestingly, FK866 treatment primarily affected phosphatidylcholine (PC) metabolism, altering 23 out of 32 species (Figure 3C, *p<0.05, DMSO versus FK866). On the other hand, within the phosphatidyl -ethanolamine (PE), -inositide (PI), and -serine (PS) classes of phospholipids, only 4 of 52 detected species were significantly changed (Figure 4A–C). Of the 23 PC species that were affected by FK866 treatment, 8 decreased and 15 increased. The PC species reduced by Nampt inhibition were 30:0 PC, 30:1 PC, 32:0 PC, 32:1 PC, 32:2 PC, 34:2 PC and the ether lipid species e32:1 PC, and e34:1 PC. Most of the decreased species were restored to control levels by exogenous NMN. The species most affected were 30:0 PC, 30:1 PC, and 32:0 PC which were reduced to 41%, 48%, and 42% of control levels, respectively. Of note, the fatty acid substituents that comprise the PC species reduced by Nampt inhibition correspond to the 14∶0, 16∶0, and 16∶1 fatty acid species that were also decreased in the FAME analysis (Figure 2D). The 15 PC species that increased with Nampt inhibition were 36:1 PC, 36:4 PC, 38:3 PC, 38:4 PC, 38:5 PC, 38:6 PC, 40:5 PC, 40:6 PC, 40:7 PC, and the ether lipid species e32:2 PC, e36:4 PC, e36:5 PC, e38:4 PC e38:5 PC, and e38:6 PC. The fatty acid substituents in the increased PC species also correspond to those that increased with FK866 treatment. Furthermore, most of the PC species that increased contained a polyunsaturated fatty acid (PUFA) in their composition. Among the PS, PE and PI classes, 40:4 PS levels increased, while 38:7 PE, 36:1 PI, and 36:2 PI levels decreased (*p<0.05). Interestingly, although 36:1 PI levels decreased, the equivalent PC species increased, and the levels of 36:2 PI decreased with no difference in the equivalent PC species. Overall, the major effects of Nampt inhibition on lipid synthesis primarily manifest through PC metabolism, suggesting that specific metabolic and signal transduction pathways may be regulated by NAD^+^ synthesis.

### Inhibition of Nampt Activates AMPK

**Figure 5:**
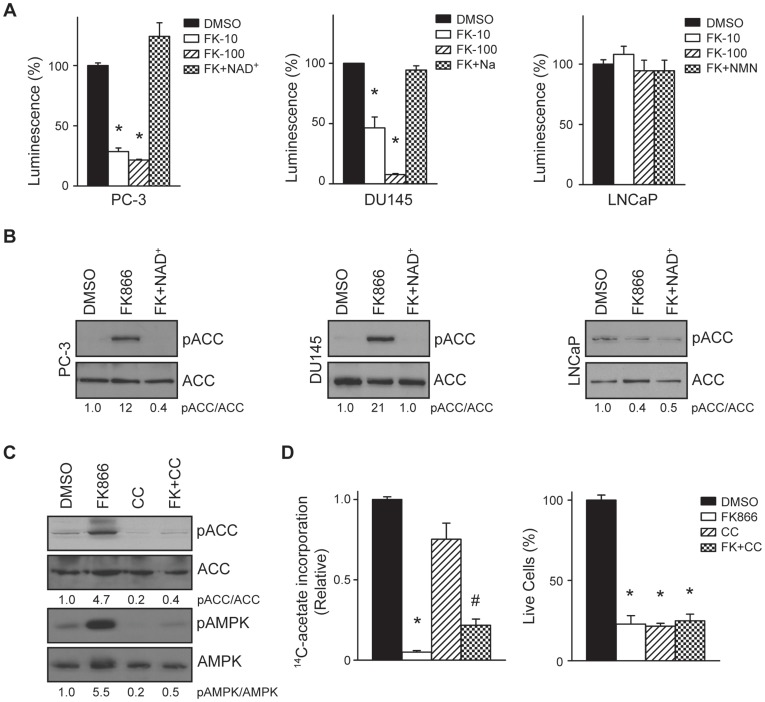
Inhibition of Nampt activates AMPK signaling (A) Prostate tumor cells were treated with vehicle, FK866 (10 nM or 100 nM), or FK866 (100 nM) plus NAD^+^, Na or NMN. After 48 hours, ATP levels were measured by luminescence and normalized to DNA content (*p<0.0001). (B) The levels of pACC and ACC were determined in cells treated with vehicle (0.1%), or FK866 (100 nM) in the absence or presence of NAD^+^ for 48 hours (100 µM). (C) PC-3 cells were treated with vehicle, FK866 (100 nM), Compound C (CC, 10 µM), or the combination of both (FK+CC) for 48 hours and pACC, ACC, pAMPK, and AMPK levels were determined by western blot. (D) PC-3 cells were treated with vehicle, FK866, Compound C, or the combination of both for 48 hours and fatty acid synthesis was measured (*p<0.0001, #p = 0.0007). Cell killing was also determined by trypan blue exclusion (*p<0.0001).

Because Nampt regulates mitochondrial function and energy homeostasis, the effect of Nampt inhibition on ATP levels and AMPK activation was ascertained. Treatment with FK866 reduced ATP in a dose-dependent manner. Treatment with 10 nM and 100 nM FK866 for 48 hours reduced ATP levels to 29% and 22% of control, respectively, in PC-3 cells and to 46% and 8% of control, respectively, in DU145 cells (*p<0.0001, Figure 5A). Although Nampt inhibition reduced NAD^+^ levels in LNCaP cells (Figure 1B), treatment with FK866 did not reduce ATP levels. Decreased ATP can result in activation of AMPK and subsequent phosphorylation and inactivation of ACC [42]. Concomitant with decreased ATP levels, Nampt inhibition increased phosphorylation of ACC at serine 79 12-fold in PC-3 cells and 21-fold in DU145 cells (Figure 5B). Phosphorylation of ACC was prevented by co-treatment with NAD^+^ in both cell lines. Consistent with our previous results, Nampt inhibition did not induce phosphorylation of ACC in LNCaP cells.

PC-3 cells were then treated with vehicle or FK866 in the absence or presence of the AMPK inhibitor Compound C. As expected, FK866 increased pACC and pAMPK levels 4.7-fold and 5.5-fold over control, respectively (Figure 5C). Co-treatment with Compound C reduced pACC and pAMPK to control levels. On the other hand, Compound C only had a modest restorative effect on fatty acid synthesis in FK866 treated cells. Compound C restored fatty acid synthesis in FK866 treated cells from 5% of control to 22% of control (*p<0.0001, DMSO vs. FK866, #p = 0.0007 FK866 vs. FK+CC, Figure 5D). Compound C was also unable to restore survival in FK866 treated cells (*p<0.0001). These data suggest that the effects of Nampt inhibition on lipogenesis correlate with AMPK activation and phosphorylation of ACC, but are also mediated by an AMPK-independent mechanism.

### Sirtuins Regulate de Novo Lipogenesis

**Figure 6:**
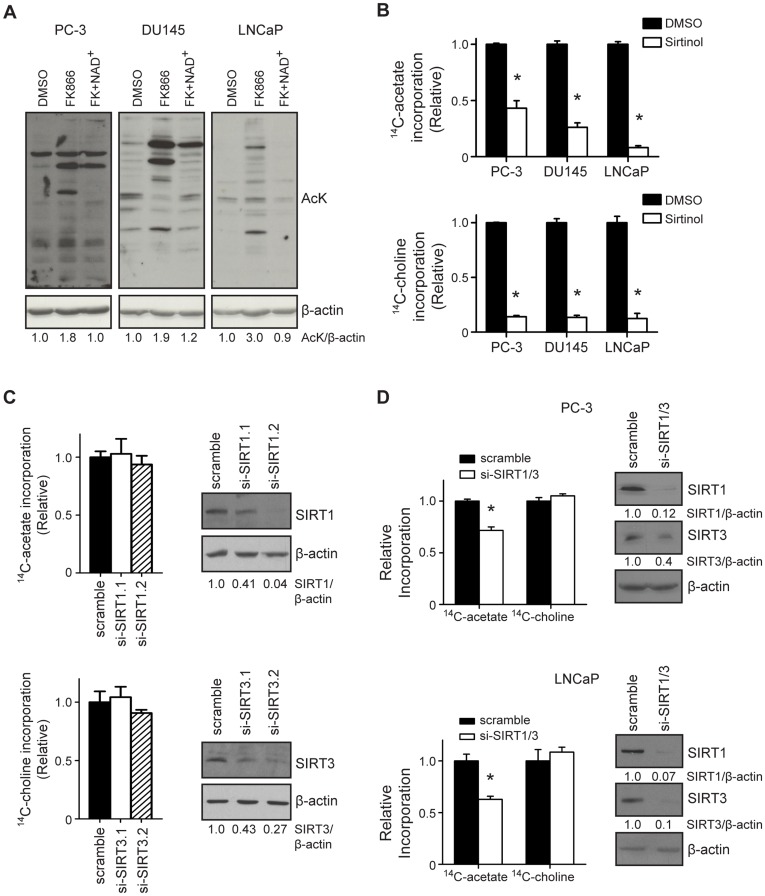
Sirtuin activity is required for lipogenesis in prostate cancer cells (A) PCa cells were treated as indicated for 48 hours and global protein acetylation was measured by western blot. (B) PCa cells were treated with DMSO (0.1%) or Sirtinol (100 µM) for 48 hours and ^14^C-acetate and -choline incorporation into lipid were measured (*p<0.0001). (C) PC-3 cells were treated with scrambled, SIRT1, or SIRT3 targeting siRNA (100 nM each) and fatty acid synthesis was measured. The expression levels of SIRT1, SIRT3, and β-actin were determined by western blot. (D) PC-3 and LNCaP cells were transfected with scrambled (200 nM) or the combination of SIRT1.2 and SIRT3.2 targeting siRNA (100 nM each). The incorporation of ^14^C-acetate and -choline into lipid were determined 5 days after transfection (*p = 0.0002). The levels of SIRT1, SIRT3, and β-actin were determined by western blot.

Nampt is required for sirtuin activity in mammalian cells [35]. In addition, SIRT1 and SIRT3 each deacetylate and activate mammalian acetyl-CoA synthetases, allowing for entry of acetate into the fatty acid synthesis pathway [23], [24]. Therefore, we reasoned that the impact of Nampt inhibition on lipogenesis would be mediated by sirtuins in tumor cells. Indeed, blockade of Nampt activity increased global protein acetylation (AcK) levels by 1.8-fold in PC-3 cells, 1.9-fold in DU145 cells, and 3-fold in LNCaP cells (Figure 6A), supporting previous observations that Nampt can regulate sirtuin function [35]. Although we were unable to detect acetylation of AceCS1 following Nampt inhibition in PCa cells (data not shown), inhibition of sirtuin activity with Sirtinol reduced ^14^C-acetate incorporation into fatty acids by 57%, 74%, and 92% in PC-3, DU145, and LNCaP cells, respectively (Figure 6B). Sirtinol also reduced the incorporation of ^14^C-choline into lipids by 86%, 87%, and 78% in PC-3, DU145, and LNCaP cells, respectively (*p<0.0001, Figure 6B). Because SIRT1 and SIRT3 are known to deacetylate and activate mammalian acetyl-CoA synthetases, fatty acid synthesis was measured following knockdown of SIRT1 and SIRT3 [23], [24]. Knockdown of SIRT1 or SIRT3 individually by siRNA did not reduce fatty acid synthesis (Figure 6C). On the other hand, simultaneous knockdown of SIRT1 and SIRT3 reduced incorporation of acetate into fatty acids by 28% in PC-3 cells, and 37% in LNCaP cells (*p = 0.0002, Figure 6D). Interestingly, choline incorporation was not reduced by SIRT1/3 knockdown. Taken together, these data suggest that the effect of Nampt inhibition on lipogenesis is conferred by decreased sirtuin activity.

## Discussion

Nampt has garnered attention as a potential therapeutic target in cancer and other metabolic diseases [17]. Nampt expression is high in several cancers and its activity is required for tumor cells to proliferate and survive [5], [8], [9], [10]. Nampt has been implicated in the control of sirtuin activity, mitochondrial function, and cellular lifespan [11], [12], [13], [35]. The metabolic pathways in cancer cells that are specifically regulated by Nampt and NAD^+^ synthesis have not been described in depth, however. Previous studies show that Nampt inhibition has no effect on glucose metabolism in tumor cells [4], [9]. The data presented above, however, detail the novel observation that Nampt is required for fatty acid and lipid synthesis in tumor cells through sirtuin-dependent mechanisms.

Pharmacologic blockade of Nampt as well as siRNA-mediated knockdown impaired lipogenesis in most cell lines. Similarly, Sirtinol treatment inhibited fatty acid and lipid synthesis in all prostate tumor cell lines tested. Additionally, simultaneous knockdown of SIRT1 and SIRT3 also reduced fatty acid synthesis in PC-3 and LNCaP cells. Taken together, these data demonstrate that Nampt and sirtuin activities are required for *de novo* lipogenesis in cancer cells. It is worth noting that the Nampt substrate nicotinamide (Nam) also inhibits sirtuins [43]. Accordingly, it is possible that Nampt inhibition could limit sirtuin activity through accumulation of Nam and reduced NAD^+^ levels. The impact of Nampt inhibition on Nam levels was not determined in our study, however. Moreover, the addition of NMN to all cell lines, and Na to DU145 cells, prevented the effects of Nampt inhibition on cell death and lipid synthesis. These data, along with the finding that knockdown of SIRT1 and 3 also inhibit lipogenesis, suggest that the effects of Nampt inhibition on sirtuin activity are primarily through depletion of NAD^+^ levels rather that accumulation of Nam.

Inhibition of Nampt induces activation of AMPK and phosphorylation of ACC. These data are consistent with the fact that NAD^+^ is required for ATP production and subsequent energy homeostasis in tumor cells. Additionally, SIRT1 and SIRT3 have both been implicated in the control of fatty acid oxidation (FAO) by deacetylation of multiple enzymes [25], [26], [27]. Because FAO has come to the forefront as an important aspect of energy control and survival in tumor cells, it is possible that Nampt inhibition also inhibits FAO, leading to activation of AMPK. Although AMPK activation and phosphorylation are important regulatory steps in fatty acid synthesis, our data demonstrate that phosphorylation is not the only step that reduces fatty acid synthesis. Pharmacological blockade of AMPK with Compound C reduced ACC phosphorylation without fully restoring fatty acid synthesis. These data illustrate that protein acetylation acts in parallel with ACC phosphorylation to limit fatty acid synthesis when Nampt activity is inhibited. Previous studies show that acetylation and phosphorylation of the transcription factors FOXO1 and STAT1 can occur simultaneously resulting in altered function [44], [45]. It is likely that that one event does not control the other, rather, that acetylation and phosphorylation serve as back-up mechanisms for one another. Nampt inhibition also reduced the levels of NADPH, an essential cofactor for FASN function. Thus, by affecting energy homeostasis, protein acetylation, and cofactor concentration, our data reveal a multi-factorial role for Nampt in the regulation of tumor cell lipogenesis.

Our data demonstrate that SIRT1 and SIRT3 coordinately regulate fatty acid synthesis in tumor cells. Because simultaneous knockdown of SIRT1 and SIRT3 was required to inhibit fatty acid synthesis, it suggests that the two enzymes have redundant functions in the pathway. This is consistent with previous findings that SIRT1 and SIRT3, but no other sirtuin family members, deacetylate AceCS1 and AceCS2 [23], [24]. Although we were unable to detect acetylation of acetyl-CoA synthetases (not shown), these data, together with previous reports, are consistent with the notion that AceCS1 and AceCS2 are acetylated when Nampt activity is blocked. However, the data do not rule out the possibility that other lipogenic enzymes are affected by Nampt inhibition. There are several examples of other protein substrates that are recognized by multiple sirtuins, including the cancer-related proteins p53, FOXO1, and NF-κB [46], [47], [48]. A redundancy mechanism also exists in breast cancer cells whereby silencing of both SIRT1 and SIRT2 is required to induce cell death [49]. As mentioned, it is also possible that other lipogenic enzymes are affected by Nampt inhibition and sirtuin blockade. For example, an analysis of the human liver protein acetylome identified ACC, the rate-limiting enzyme of fatty acid synthesis, and FASN, the enzyme that synthesizes fatty acids, as potential targets for acetylation [22]. This suggests that fatty acid synthesis may be regulated at multiple levels by acetylation. It is interesting that Nampt blockade did not affect fatty acid synthesis in LNCaP cells. Treatment with FK866 reduced total NAD^+^ levels in LNCaP cells as efficiently as in PC-3 and DU145 cells, without reducing ATP levels or fatty acid synthesis. On the other hand, Sirtinol treatment and SIRT1/3 knockdown did reduce fatty acid synthesis. These data demonstrate that NAD^+^ metabolism is important for lipogenesis in cancer cells. There is at least one report demonstrating that in some cell lines mitochondrial NAD^+^ levels are insensitive to the effects of FK866. Thus, it is possible that mitochondria in LNCaP cells are relatively unaffected by FK866. This would explain why ATP levels and fatty acid synthesis would not be decreased by FK866 in LNCaP cells. Some cell lines also appear to not express Nampt in the mitochondria, making them less susceptible to Nampt inhibition, although this is not the case in LNCaP cells [50]. Deciphering the precise mechanism by which fatty acid synthesis is regulated by NAD^+^ in tumor cells will lead to a greater understanding of the complex mechanisms of tumor cell metabolism, and how NAD^+^ metabolism contributes to cancer pathogenesis.

The effect of Nampt inhibition on lipid metabolism is especially evident within the PC class of glycerophospholipids where there was an overall shift from higher levels of saturated and mono-unsaturated PC species to a composition enriched in poly-unsaturated species. This is similar to the effects on the lipid profile of cells in which FASN and ACC activities have been inhibited [51], [52]. A distinction of these data is that the effect of Nampt inhibition is primarily concentrated with the PC class of lipids, whereas FASN and ACC inhibition also impact the other lipid classes. A previous study also demonstrated Nampt inhibition results in decreased phosphocholine levels, suggesting the possibility that choline kinase may be inhibited [53]. It is intriguing to speculate that choline kinase may be acetylated when Nampt is inhibited, although we were unable to detect choline kinase acetylation in lysates from cells treated with FK866 (not shown). There is also evidence that choline-phosphate cytidylyltransferase 1β in the Kennedy pathway of PC synthesis is acetylated in human liver tissue samples [22]. The data presented in this study, along with previous reports, demonstrates that PC synthesis also appears to be subject to metabolic regulation by Nampt and acetylation. We demonstrate that fatty acid synthesis can be inhibited by Sirtinol and by silencing of SIRT1 and SIRT3, whereas lipid synthesis was only blocked by Sirtinol but not SIRT1/3 knockdown. The data demonstrate that fatty acid and lipid synthesis are differentially regulated by multiple sirtuins, which likely have redundant functions. It is also worth noting that Nampt inhibition reduced lipid synthesis in LNCaP cells, but not fatty acid synthesis. Because LNCaP cells did not activate AMPK or phosphorylate ACC when Nampt was inhibited, the effects on lipid metabolism appear to be independent of AMPK. This further supports the notion the PC metabolizing enzymes may be acetylated when Nampt is inhibited.

Collectively, we have identified a novel connection between Nampt, NAD^+^ metabolism, and lipogenesis. Because of the detrimental side effects of standard therapies for the treatment of PCa, Nampt blockade could serve as a novel therapeutic strategy to inhibit fatty acid synthesis and induce tumor cell death, while preserving normal organ function. FK866 has undergone a Phase I clinical trial for solid malignancies and several NAD^+^ synthesis inhibitors are also currently under development [16], [54], [55], [56]. Sirtuin inhibitors also have promise as anti-cancer drugs [57], [58], [59], [60]. Additionally, AMPK activators also have promise against multiple cancers. Because Nampt inhibition affects AMPK and sirtuins, Nampt inhibitors could yield an efficient mode of blocking tumor growth. This growing preclinical evidence, combined with the development of new and effective Nampt inhibitors, could provide a novel avenue for metabolic reprogramming and enhance strategies for cancer therapy.
